# The Maidstone Epidemic

**Published:** 1898-01-08

**Authors:** 


### THE MAIDSTONE EPIDEMIC.

The report by Mr. Adams and Dr. Washbourn upon the water supply of Maidstone was issued last Saturday, and serves well to indicate the limits which must be placed upon all our deductions from the results of chemical and bacteriological examinations of suspected waters. Tracing the history of the epidemic backwards it is indeed difficult to doubt that, although when once the virus was distributed a great deal of secondary typhoid arose, the original distribution of the infective material took place from certain gathering grounds in the Farleigh area. But on examination of these waters no typhoid bacilli could be found, and Dr. Washbourn has done well to state definitely that "it is a debateable question whether it is possible in the present state of our knowledge to discover the presence of the typhoid bacillus in water contaminated with animal material. . . . The difficulty, if not impossibility, of recognising the typhoid bacillus in these circumstances (*i.e.*, in water polluted by typhoid excreta) is so great that no importance can be attached to the failure of discovering the typhoid bacillus in water suspected of being the cause of an epidemic of typhoid fever."

If this is the present position of bacteriological science, what are we to think of the stress which (in the reports of the Local Government Board on the London water) is laid upon the fact that "the most minute microscopic examination of the water as it reaches the intakes of the various companies has hitherto failed to discover in it a single pathogenic germ"?

Clearly such reports must be read with knowledge. In this connection we would point out that while in the Tutsham-in-Field spring water, which is described as "thoroughly bad," 560 bacteria per cubic centimetre were discovered, the number of microbes in the Thames water at Hampton is given in the last report of the Local Government Board as ranging from 1,740 per c.c. in August to 160,000 per c.c. in December, and that even after filtration the water delivered to Londoners has been known on certain occasions to contain 3,034, and even 16,000, microbes per c.c. In the face of such facts as these, it is clear that it is unsafe to rely much upon any tests of water purity which depend on mere microbe counting; and it becomes a serious question whether even now we must not go back to the extreme position taken by Sir George Buchanan, who, in his evidence before a Royal Commission, said that, if we wanted pure water, we must "go and see that what ought not to get into water does not get into it."

